# Evidence of cross-cutting and redox reaction in Khatyrka meteorite reveals metallic-Al minerals formed in outer space

**DOI:** 10.1038/s41598-017-01445-5

**Published:** 2017-05-09

**Authors:** Chaney Lin, Lincoln S. Hollister, Glenn J. MacPherson, Luca Bindi, Chi Ma, Christopher L. Andronicos, Paul J. Steinhardt

**Affiliations:** 1 Department of Physics, Princeton University, Jadwin Hall, Princeton, NJ 08544 USA; 2 Department of Geosciences, Princeton University, Guyot Hall, Princeton, NJ 08544 USA; 3 Department of Mineral Sciences, National Museum of Natural History, Smithsonian Institution, Washington DC, 20560 USA; 4 Dipartimento di Scienze della Terra, Università di Firenze, Via La Pira 4, I-50121 Florence, Italy; 5 C.N.R. – Istituto di Geoscienze e Georisorse, Via La Pira 4, I-50121 Florence, Italy; 6 Division of Geological and Planetary Sciences, California Institute of Technology, Pasadena, CA 91125 USA; 7 Department of Earth, Atmospheric, and Planetary Sciences, Purdue University, West Lafayette, IN 47907 USA; 8 Princeton Center for Theoretical Science, Princeton University, Princeton, NJ 08544 USA

## Abstract

We report on a fragment of the quasicrystal-bearing CV3 carbonaceous chondrite Khatyrka recovered from fine-grained, clay-rich sediments in the Koryak Mountains, Chukotka (Russia). We show higher melting-point silicate glass cross-cutting lower melting-point Al-Cu-Fe alloys, as well as unambiguous evidence of a reduction-oxidation reaction history between Al-Cu-Fe alloys and silicate melt. The redox reactions involve reduction of FeO and SiO_2_ to Fe and Fe-Si metal, and oxidation of metallic Al to Al_2_O_3_, occurring where silicate melt was in contact with Al-Cu-Fe alloys. In the reaction zone, there are metallic Fe and Fe-Si beads, aluminous spinel rinds on the Al-Cu-Fe alloys, and Al_2_O_3_ enrichment in the silicate melt surrounding the alloys. From this and other evidence, we demonstrate that Khatyrka must have experienced at least two distinct events: first, an event as early as 4.564 Ga in which the first Al-Cu-Fe alloys formed; and, second, a more recent impact-induced shock in space that led to transformations of and reactions between the alloys and the meteorite matrix. The new evidence firmly establishes that the Al-Cu-Fe alloys (including quasicrystals) formed in outer space in a complex, multi-stage process.

## Introduction

The Khatyrka meteorite^1, 2^, an oxidized-subgroup (Allende-like) CV3 carbonaceous chondrite, contains silicates and oxides that are typical of CV3 (ox) chondrites^3–7^. This includes: (1) a highly porous matrix, dominated by platy olivines with ferroan compositions (Fo_50–56_) typical of CV3 chondrites^5^; (2) Type IA porphyritic olivine chondrules, with minor element variations typical of CV3 chondrule olivines^8^; and (3) calcium-aluminum-rich inclusions (CAIs) that are identical to those in oxidized CV3 chondrites^9^. However, the Khatyrka meteorite is the only meteorite known to contain quasicrystals^1, 10–14^ and metallic Al alloys^1, 10–17^. The unexpected occurrence of these phases in nature has motivated a multidisciplinary effort to understand the physical and chemical history of the Khatyrka meteorite for the purpose of revealing heretofore-unknown conditions and physical processes in the solar nebula and providing insights into the formation and stability of quasicrystals in nature.

To date, all identified material from the Khatyrka meteorite occurs as grains a few millimeters or less in diameter. Most grains were found among bags of clay recovered from a variety of depositional environments (i.e., lacustrine, alluvial, reworked) along the Listvenitovyi stream in the Koryak Mountains during a 2011 expedition to the Chukotka Autonomous Okrug in far eastern Russia^2, 11, 12^, including samples recovered from >~7000 year-old undisturbed alluvium^2^, as documented by the authors of this study (G.J.M., L.B., C.L.A., P.J.S.) who were members of the expedition. The silicates and oxides in Khatyrka are verifiably meteoritic, as supported by textural evidence, mineral chemistry, oxygen isotopic measurements^2^ and measurements of noble gas cosmogenic nuclides^18, 19^.

Despite this abundant evidence, the difficulty in interpreting the Al-Cu alloys as being of natural meteoritic origin^1, 2^ is twofold: (i) metallic Al requires extremely reducing conditions to form—even the highly-reducing innermost hot regions of the pre-solar nebula at 4.567 Ga^20^ were nowhere near sufficiently reducing to stabilize pure, metallic Al; and (ii) Al and Cu have greatly differing cosmochemical behaviors: Al is a refractory lithophile element that condenses at very high temperatures out of a hot and cooling gas of Solar composition, whereas Cu is a moderately volatile siderophile/chalcophile element that condenses at a much lower temperature than Al. A complicating factor has been that, until now, there were no clear signs of chemical reaction between the reduced metal alloys and the oxidized meteoritic silicates. The observations presented in this paper correct this situation.

To be sure of the natural meteoritic origin, it is critical to have clear evidence of chemical reactions between the reduced Al-Cu-Fe metal alloys and the oxidized meteoritic silicates. This paper provides that evidence. This new evidence is from a recently identified fragment of Grain 126, found during our continued study of grains from the Khatyrka meteorite. To distinguish the fragment from others of Grain 126, we refer to it as “Grain 126A”. It is deposited at the Smithsonian Institution’s National Museum of Natural History, Washington DC, USA under the catalogue number USNM 7908. We also present evidence of a time sequence between the Al-Cu-Fe metal phases and silicate matrix, and document the petrological context of four new Al-Cu-Fe minerals, discovered in Grain 126A and recently reported in refs 14, 16 and 17. We note that the interpretation here of a reaction history is supported by preliminary studies on Grain 129, some results of which were reported in ref. 21.

Earlier investigations of Khatyrka have produced abundant petrologic and chemical evidence showing that Khatyrka experienced at least one high-velocity impact event^21, 22^. This includes the observation of two high-pressure Fe-bearing phases: ahrensite (Fe_2_SiO_4_)^23^ and an unnamed oxide with composition Fe_2.6_Si_0.4_O_4_ that has the structure of spinelloid V^24^. Measurements of noble gas cosmogenic nuclides in the olivine of 126 show that the most recent major impact event experienced by Khatyrka occurred in space a few 100 Ma^18, 19^ and produced shocks consistent with the range S3 to S5 (though probably closer to S4), corresponding to approximately 10–35 GPa^25^. An impact of this magnitude could account for the high-pressure phases, as well as the silicate melt and reactions that we report herein. Because there is currently no evidence to the contrary, we will assume this is the case for the purpose of this discussion, referring only to a single ‘impact event’ occurring a few 100 Ma, while acknowledging that we could also be observing the results of a series of impact events occurring over eons of time.

Based on the evidence of impact, two competing hypotheses were proposed in ref. 22 to explain the relationship between the ‘impact event’ that led to the formation of high-pressure FeO-bearing phases and the event that formed the Al-Cu-Fe metal, which we refer to as the ‘metal-forming event’.

In the first hypothesis, the ‘impact event’ and ‘metal-forming event’ are the same. In this hypothesis, the Al-Cu-Fe alloys did not exist prior to the impact; instead, the Al, Cu, and Fe were incorporated in other phases, possibly Al- and Cu-bearing Fe-Ni metals, which then melted upon impact. Post-shock liberation of Cu has been discussed in the literature^26, 27^, and Cu-bearing metal phases are commonly observed in meteorites (see, e.g., ref. 26). The Al would be similarly exsolved; however, Al-bearing Fe-Ni metals have only been observed and extensively studied in Khatyrka^13, 15^, although an unconfirmed example has also been noted in a study of the shocked Suizhou L6 chondrite^28^. A challenge for this hypothesis is to explain the amount of Al and Cu that would have to be liberated to account for the observed volume of Al-Cu alloys in Khatyrka.

In the second hypothesis, the ‘impact event’ and ‘metal-forming event’ are not the same. The essential difference is that, according to this hypothesis, whatever event formed the Al-Cu-Fe metals is different from the impact event(s) that produced the observed high-pressure FeO-bearing mineral phases. We remark that this hypothesis, as stated, does not make any claims as to the specific mechanism for how the first Al-Cu-Fe alloys formed.

Here, we will present evidence of cross-cutting relationships and redox reactions that support the second hypothesis and provide clues about the sequence of events that occurred in 126A during the more recent impact a few 100 Ma. The results indicate that some, but not most, of the Al-bearing grains observed in 126 A (including some quasicrystals) were formed as a direct result of the impact in space. An important corollary is that most of the Al-bearing alloys (including quasicrystals) existed prior to the impact, and so had to have formed in space at some earlier time.

## Results

### Description of sample

**Figure 1:**
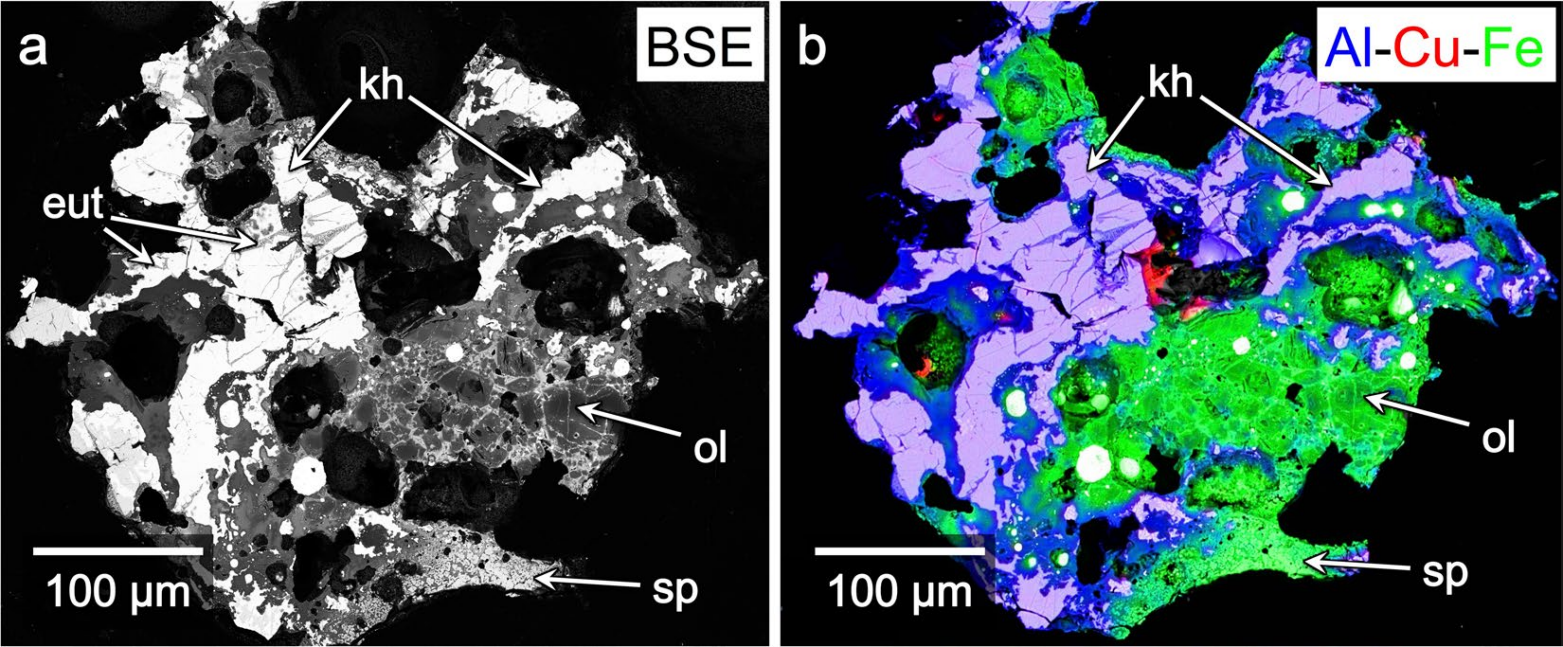
Overview of Khatyrka Grain 126A. (**a**) Backscattered electron (BSE) image of Grain 126A. Bright regions are mostly Al-Cu-Fe metal assemblages; they have an irregular, cuspate-lobate appearance and consist predominantly of khatyrkite (“kh”), stolperite, and eutectoid regions (“eut”; further detail in Fig. 3) that contain a vermicular mixture of metallic Al (up to 13.3 weight% Cu) and khatyrkite. The darker regions mostly comprise crystals of olivine (“ol”; further detail in Fig. 2c and d) and spinel-group minerals with varying composition, which we call “spinel” (“sp”; further detail in Fig. 6)—all surrounded by silicate glass. (**b**) Al-Cu-Fe combined X-ray area map, overlaid on a BSE image. Light purple regions are Al-Cu metal (khatyrkite, stolperite); blue/dark purple regions are predominantly glass and spinel; green regions are mainly the silicate glass and crystals that grew within the melt (olivine, spinel); the relatively large white grains are predominantly Fe-Ni (appearing white because of the underlying BSE image, despite containing Fe). The different compositions of spinel manifest here as different degrees of brightness (**a**) and different colors (**b**).

Grain 126A (Fig. 1) is dominated by relatively large (100–300 μm), irregularly shaped assemblages of Al-Cu-Fe metals (lighter, Fig. 1a; blue/purple, Fig. 1b) and, in the center-right of the figure, irregularly shaped olivine grains (10–30 μm). There are also smaller Al-Cu-Fe metal grains (5–20 μm), Fe-Ni globules (3–25 μm, round and bright in Fig. 1a and b), and a fine-grained, hypohyaline assemblage of olivine (2–10 μm) and spinel-group (<2 μm) crystals. (We refer to the spinel-group minerals, which occur in 126A with various compositions, as “spinel”.) Each Al-Cu-Fe metal assemblage is surrounded by a rim of spinel crystals (<1 μm). Most assemblages have, adjacent to this spinel layer, Fe and Fe-Si metal beads, varying in size from <10 nm to ~5 μm. All of these phases are set in a glass. Small fragments of Al-Cu-Fe assemblages occur immediately above and below the region dominated by olivine, in the center-right of Fig. 1. The upper half and the left half of the figure are dominated by the relatively large Al-Cu-Fe assemblages.

**Figure 2:**
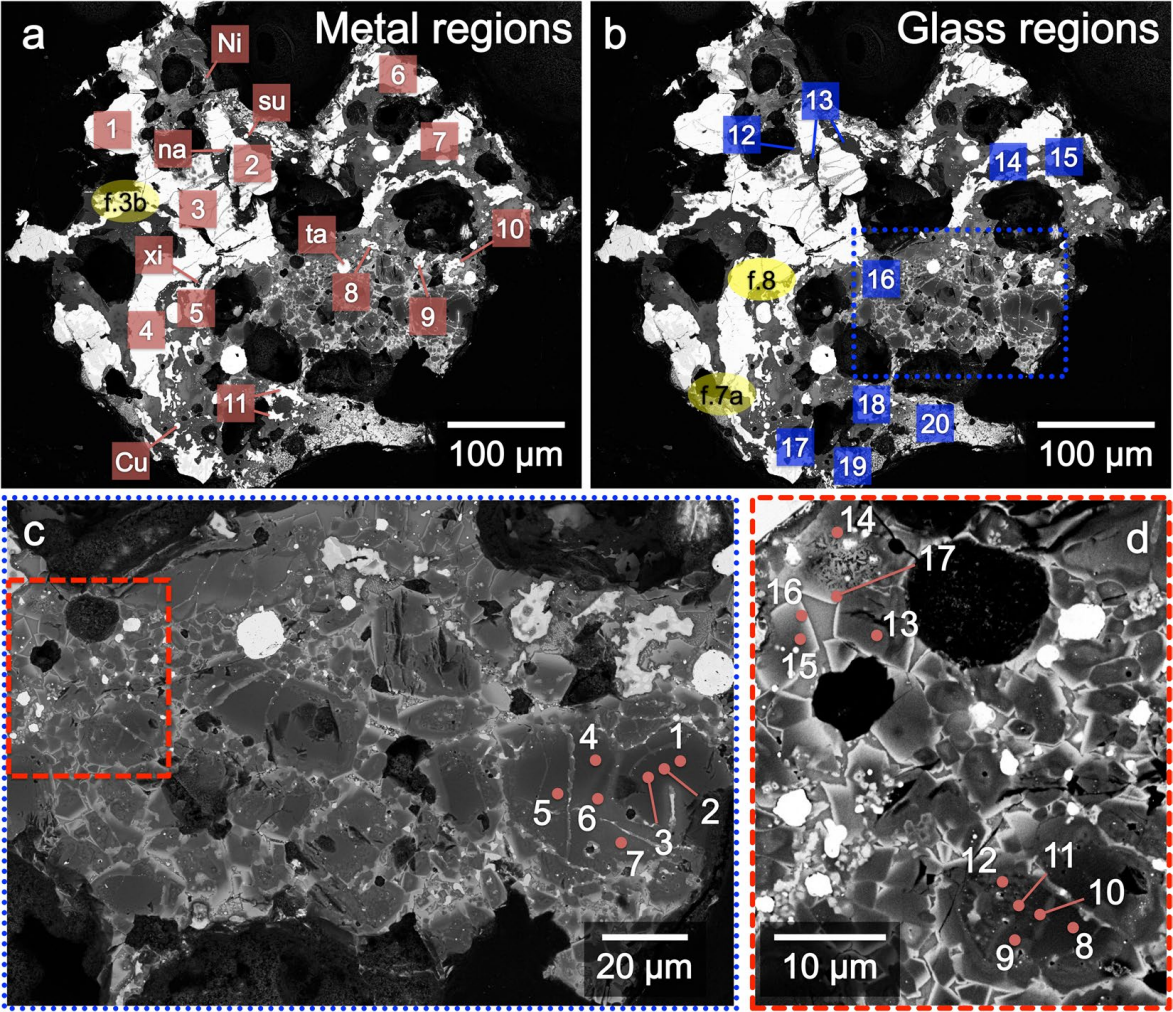
BSE images, marked to show locations of EPMA-WDS and SEM-EDS analyses, with close up views of olivine in (**c**) and (**d**). Red squares in (**a**) indicate metal regions referred to in Table 1. Blue squares in (**b**) indicate glass regions referred to in Table 2b and Fig. 5. Yellow ovals in (**a**) and (**b**) are referred to in Figs 3b, 7a, and 8. (**c**) Close up of area boxed in blue in (**b**). This region contains several olivine crystals. The larger grains are cross-cut by Fe-rich veins that are continuously connected to glass. Analyses of numbered spots are provided in Table 2a. These appear to be relict olivine grains. (**d**) Close up of area boxed in red in (**c**) showing small euhedral to subhedral olivine grains that appear to be a non-relict, second generation of olivine that grew from the matrix melt. Analyses of numbered spots are provided in Table 2a.

Averaged and representative analyses of the different phases are presented in Tables 1 and 2; regions that were analyzed are shown in Fig. 2. For the olivine and spinel compositions, we took care to center the electron beam on individual crystals to minimize excitation of elements in neighboring phases. Similarly, for the glass compositions, we took care to avoid crystalline material. Electron backscatter diffraction was used to identify the structure of various phases.

**Table 1:** Averaged elemental compositions of metal phases in 126A by EPMA-WDS. All in elemental weight %.

Phase	Location^a^	*n*	Al	Fe	Cu	Ni	Si	Mg	Ca	Cr	Total
khatyrkite	1	16	47.89 (17)	0.54 (14)	51.22 (51)	b.d.l.	b.d.l.	b.d.l.	b.d.l.	b.d.l.	99.65
	2	9	48.01 (27)	0.72 (20)	51.73 (48)	b.d.l.	b.d.l.	b.d.l.	b.d.l.	b.d.l.	100.46
	3	13	47.70 (28)	0.61 (14)	51.34 (54)	b.d.l.	b.d.l.	b.d.l.	b.d.l.	b.d.l.	99.65
	4	9	47.97 (43)	1.04 (6)	50.97 (51)	b.d.l.	0.06 (2)	b.d.l.	b.d.l.	b.d.l.	100.04
	5	5	48.88 (49)	1.40 (12)	50.07 (78)	b.d.l.	b.d.l.	b.d.l.	b.d.l.	b.d.l.	100.35
	6	8	48.16 (24)	0.85 (13)	50.94 (49)	b.d.l.	b.d.l.	b.d.l.	b.d.l.	b.d.l.	99.95
	7	11	48.06 (42)	1.34 (18)	50.78 (87)	b.d.l.	b.d.l.	b.d.l.	0.03 (2)	b.d.l.	100.21
	8	2	47.82 (19)	2.68 (1)	49.31 (3)	b.d.l.	0.16 (1)	b.d.l.	0.03 (2)	0.07 (3)	100.08
	9	2	48.74 (50)	2.10 (3)	49.94 (9)	b.d.l.	0.09 (5)	0.07 (10)	b.d.l.	b.d.l.	100.94
	10	2	48.42 (5)	2.16 (7)	49.37 (91)	b.d.l.	0.10 (2)	b.d.l.	0.05 (4)	b.d.l.	100.10
	11	2	47.24 (11)	1.55 (15)	50.20 (3)	b.d.l.	0.20 (11)	b.d.l.	b.d.l.	b.d.l.	99.19
stolperite	4	3	38.71 (20)	1.45 (12)	59.53 (59)	b.d.l.	b.d.l.	b.d.l.	0.03 (1)	b.d.l.	99.72
	5	3	38.33 (11)	2.70 (32)	57.77 (46)	b.d.l.	b.d.l.	b.d.l.	b.d.l.	0.05 (3)	98.85
	8	2	35.94 (37)	4.23 (4)	61.23 (42)	b.d.l.	b.d.l.	b.d.l.	b.d.l.	0.07 (2)	101.47
	9	3	33.92 (8)	3.09 (11)	62.71 (62)	b.d.l.	b.d.l.	b.d.l.	b.d.l.	0.05 (1)	99.78
	10	2	35.80 (5)	2.65 (26)	60.80 (11)	b.d.l.	b.d.l.	b.d.l.	0.04 (1)	b.d.l.	99.29
	11	2	36.13 (29)	2.73 (9)	60.83 (86)	b.d.l.	b.d.l.	b.d.l.	b.d.l.	b.d.l.	99.69
*i*-I	8	3	43.16 (13)	15.04 (50)	41.03 (41)	b.d.l.	0.14 (1)	b.d.l.	0.04 (1)	0.14 (1)	99.55
*i*-II	9	3	41.98 (67)	9.43 (94)	47.26 (92)	b.d.l.	0.08 (1)	0.04 (6)	b.d.l.	0.09 (3)	98.88
*i*-II	10	9	40.32 (47)	9.16 (35)	48.74 (64)	b.d.l.	0.06 (3)	0.06 (6)	0.04 (1)	0.11 (2)	98.49
kryachkoite	4	3	59.95 (65)	11.80 (14)	26.38 (30)	b.d.l.	0.16 (3)	b.d.l.	b.d.l.	0.32 (2)	98.61
hollisterite	8	4	55.04 (40)	30.42 (64)	14.15 (25)	b.d.l.	0.30 (1)	b.d.l.	0.03 (1)	0.16 (3)	100.11
iron^b^		5	b.d.l.	95.02 (52)	1.02 (13)	2.04 (9)	b.d.l.	n.a.	n.a.	n.a.	98.08
suessite	su	3	b.d.l.	80.89 (61)	1.85 (14)	0.55 (4)	14.23 (19)	n.a.	n.a.	n.a.	97.52
naquite	na	3	0.83 (38)	57.22 (64)	2.09 (39)	0.73 (4)	35.33 (19)	0.31 (12)	0.09 (3)	1.06 (1)	97.66
nickel	Ni	3	b.d.l.	3.86 (11)	3.53 (15)	90.45 (43)	b.d.l.	b.d.l.	b.d.l.	b.d.l.	97.84
copper	Cu	2	b.d.l.	3.82 (21)	94.19 (29)	b.d.l.	b.d.l.	n.a.	n.a.	n.a.	98.01
taenite	ta	4	b.d.l.	66.03 (40)	0.67 (9)	30.36 (30)	b.d.l.	n.a.	n.a.	n.a.	97.06
xifengite^c^	xi	1	b.d.l.	72.27 (42)	2.10 (43)	b.d.l.	24.46 (27)	b.d.l.	b.d.l.	1.17 (14)	100.00

*n*: number of analyses included in average, n.a.: not analyzed, b.d.l.: below detection limits, 0.07 wt% Al, 0.2% Ni, 0.05% Si, 0.04% Mg, 0.03% Ca, 0.05% Cr; SEM-EDS detection limit ~0.1 wt%. Uncertainty given in parentheses represents one standard deviation from the mean based on all analyses [e.g., 50.3 (13) = 50.3 +/− 1.3]. ^a^Location refers to Fig. 2a. ^b^Iron composition is averaged over multiple iron beads. ^c^Xifengite analyzed by SEM-EDS (normalized total to 100%).

**Table 2:** Point analyses of olivine (a) and averaged compositions of glass (b) and spinel (c) in 126A by EPMA-WDS. All in compound weight %.

a. *Olivine*													
Spot^a^	Fo^b^	SiO_2_	Al_2_O_3_	FeO	Fe_2_O_3_	MgO	CaO	Na_2_O	P_2_O_5_	CuO	Cr_2_O_3_	NiO	Total
S1	98	42.7 (2)	0.11 (2)	1.45 (8)	-	55.5 (2)	0.61 (2)	b.d.l.	b.d.l.	b.d.l.	0.18 (5)	b.d.l.	100.55
S2	98	42.7 (2)	0.14 (2)	1.23 (7)		55.4 (2)	0.65 (2)	b.d.l.	b.d.l.	b.d.l.	0.18 (5)	b.d.l.	100.30
S3	99	42.7 (2)	0.17 (2)	1.00 (7)		56.3 (2)	0.66 (2)	b.d.l.	b.d.l.	b.d.l.	0.13 (5)	b.d.l.	100.96
S4	95	41.7 (2)	0.11 (2)	5.0 (1)		53.3 (2)	0.57 (2)	b.d.l.	b.d.l.	0.09 (4)	0.21 (5)	b.d.l.	100.98
S5	92	41.1 (2)	0.09 (2)	8.0 (2)		50.4 (2)	0.49 (1)	b.d.l.	b.d.l.	0.11 (4)	0.15 (5)	b.d.l.	100.34
S6	84	39.9 (2)	0.07 (2)	14.9 (2)		43.9 (2)	0.56 (2)	0.03 (2)	b.d.l.	b.d.l.	0.13 (5)	b.d.l.	99.49
S7	77	38.8 (2)	0.05 (2)	21.2 (3)		39.6 (2)	0.39 (1)	b.d.l.	b.d.l.	b.d.l.	b.d.l.	0.08 (4)	100.12
S8	88	40.6 (2)	0.11 (2)	11.1 (2)		47.6 (2)	0.25 (1)	b.d.l.	b.d.l.	0.19 (4)	0.10 (5)	b.d.l.	99.95
S9	85	39.5 (2)	0.10 (2)	14.3 (2)		44.9 (2)	0.27 (1)	0.05 (2)	b.d.l.	0.16 (4)	b.d.l.	0.27 (4)	99.55
S10	79	39.0 (2)	0.06 (2)	18.1 (2)		40.6 (2)	0.23 (1)	b.d.l.	b.d.l.	0.25 (5)	0.21 (5)	1.83 (6)	100.28
S11	82	39.5 (2)	0.10 (2)	16.7 (2)		43.2 (2)	0.44 (1)	b.d.l.	b.d.l.	0.20 (5)	0.13 (5)	0.11 (4)	100.38
S12	78	37.9 (1)	1.12 (3)	19.6 (2)		40.4 (2)	0.25 (1)	b.d.l.	0.06 (3)	0.15 (5)	0.36 (6)	0.67 (4)	100.51
S13	73	37.6 (1)	0.80 (3)	22.9 (3)		36.1 (2)	0.17 (1)	b.d.l.	0.73 (5)	0.44 (5)	0.35 (6)	b.d.l.	99.09
S14	64	36.4 (1)	0.98 (3)	29.9 (3)		29.9 (1)	0.18 (1)	b.d.l.	0.82 (5)	0.80 (5)	0.14 (5)	b.d.l.	99.12
S15	61	35.4 (1)	0.97 (3)	32.0 (3)		28.7 (1)	0.17 (1)	b.d.l.	0.97 (5)	0.62 (5)	0.11 (5)	0.08 (4)	99.02
S16	45	33.6 (1)	0.87 (3)	43.6 (4)		20.1 (1)	0.31 (1)	b.d.l.	1.07 (6)	0.83 (6)	0.11 (4)	b.d.l.	100.49
S17	57	35.2 (1)	0.69 (3)	35.4 (3)		26.4 (1)	0.18 (1)	b.d.l.	0.86 (5)	0.67 (5)	b.d.l.	b.d.l.	99.40
**b**. ***Glass***													
**Location** ^**c**^	***n***	**SiO**_**2**_	**Al**_**2**_ **O**_**3**_	**FeO** ^**d**^	**Fe**_**2**_ **O**_**3**_	**MgO**	**CaO**	**Na**_**2**_ **O**	**P**_**2**_ **O**_**5**_	**CuO**	**Cr**_**2**_ **O**_**3**_	**NiO**	**Total**
12	3	40.7 (42)	15.4 (29)	10.9 (10)	-	16.66 (32)	9.0 (11)	1.263 (73)	0.350 (60)	1.403 (27)	b.d.l.	0.21 (10)	95.90
13	6	46.57 (66)	28.4 (22)	0.60 (26)		14.9 (27)	7.47 (62)	1.07 (32)	b.d.l.	0.897 (85)	b.d.l.	b.d.l.	99.96
14	4	44.9 (21)	22.9 (59)	9.0 (66)		15.6 (27)	4.25 (65)	1.37 (78)	0.17 (17)	0.87 (10)	0.12 (10)	b.d.l.	99.12
15	4	42.9 (34)	16.49 (66)	23.8 (35)		8.1 (22)	4.10 (41)	0.41 (10)	0.54 (11)	0.83 (25)	b.d.l.	b.d.l.	97.20
16	2	35.2 (46)	18.4 (12)	35.11 (79)		3.1 (28)	5.6 (14)	0.450 (43)	1.63 (36)	0.80 (35)	b.d.l.	b.d.l.	100.30
17	19	34 (10)	30 (10)	15.7 (81)		13.9 (23)	1.94 (68)	0.38 (22)	0.54 (74)	0.68 (32)	0.63 (54)	b.d.l.	97.80
18	6	45.94 (49)	32.07 (78)	4.53 (88)		12.7 (12)	2.862 (76)	0.40 (12)	b.d.l.	0.626 (59)	0.302 (53)	b.d.l.	99.42
19	4	42.0 (51)	15.3 (31)	18.8 (14)		13.7 (15)	2.39 (23)	0.264 (27)	0.68 (13)	0.593 (53)	0.14 (23)	b.d.l.	93.84
20	3	36.3 (38)	4.7 (36)	33.8 (38)		13.5 (75)	2.0 (18)	0.47 (59)	1.19 (58)	0.38 (25)	b.d.l.	2.9 (38)	95.31
**c**. ***Spinel***													
**Phase**	***n***	**SiO**_**2**_	**Al**_**2**_ **O**_**3**_	**FeO**	**Fe**_**2**_ **O**_**3**_^**e**^	**MgO**	**CaO**	**Na**_**2**_ **O**	**P**_**2**_ **O**_**5**_	**CuO**	**Cr**_**2**_ **O**_**3**_	**NiO**	**Total**
Crust	11	6.53 (5.86)	52.28 (13.89)	17.14 (4.73)	3.78 (9.37)	14.73 (2.56)	0.50 (0.42)	0.22 (0.14)	0.15 (0.09)	1.95 (2.17)	2.19 (1.60)	0.09 (0.01)	99.56
Sp-Al	15	3.60 (4.97)	39.87 (14.91)	21.08 (3.35)	18.85 (17.87)	12.30 (3.38)	0.25 (0.31)	0.06 (0.07)	0.16 (0.08)	0.14 (0.27)	2.53 (1.53)	2.74 (2.52)	101.57
Sp-Fe	17	3.53 (3.43)	12.81 (7.26)	25.01 (4.62)	48.41 (10.86)	6.56 (2.49)	0.24 (0.27)	0.10 (0.09)	0.11 (0.04)	0.09 (0.30)	0.64 (0.89)	4.10 (1.80)	101.61
Sp-Mg^f^	11	8.93 (4.16)	56.12 (6.25)	11.91 (4.55)	0.81 (2.03)	19.70 (2.45)	b.d.l.	b.d.l.	b.d.l.	b.d.l.	2.38 (5.23)	b.d.l.	99.85

*n*: number of analyses included in average, b.d.l.: below detection limits, 0.02 wt% Ca, 0.03% Na, 0.02% P, 0.08% Cu, 0.06% Cr, 0.06% Ni; SEM-EDS detection limit ~0.1 wt%. Uncertainty given in parentheses represents one standard deviation from the mean based on: counting statistics (for olivine); all analyses (for spinel and glass), [e.g., 50.3 (13) = 50.3 (1.3) = 50.3 +/- 1.3]. ^a^S# refers to a point analysis of olivine, where the # corresponds to a location specified in Figures 2c or 2d. ^b^Forsterite content (“Fo”) calculated as average of [Mg]/2 and 1 - [Fe]/2, where [Mg], [Fe] are atomic ratios of Mg and Fe, respectively, normalized to 3 cations. ^c^Location refers to Figure 2b. ^d^All Fe in glass compositions considered as FeO. ^e^Fe_2_O_3_ content calculated by charge balancing on the basis of 4 Oxygen atoms. ^f^Sp-Mg analyzed by SEM-EDS (unnormalized).

### Al-Cu-Fe metals

The Al-Cu-Fe metal grains have complex grain boundaries. Many of the grain boundaries display cuspate-lobate fold morphology, others are rounded to amoeboid, and some are straight and angular. In many cases, the cuspate-lobate boundaries show the cusps point to the metal grains. There is a halo of Al_2_O_3_-enrichment (appears as blue/purple, Fig. 1b), extending beyond the metal boundaries into the silicate regions. Averaged compositions of metal phases from regions marked in Fig. 2a are presented in Table 1.

Among the metal grains are four new Al-Cu-Fe minerals, which were recently reported in refs 14, 16 and 17. They include stolperite (AlCu)^16, 17^, kryachkoite ((Al,Cu)_6_(Fe,Cu))^16, 17^, hollisterite (Al_3_Fe)^16, 17^, and an as-yet-unnamed quasicrystal^14^ (denoted ‘*i*-II’*)*. The quasicrystal *i*-II has the same icosahedral symmetry as icosahedrite^1, 10^ (denoted ‘*i*-I’) but a composition Al_62.0(8)_Cu_31.2(8)_Fe_6.8(4)_, which is outside the measured equilibrium stability field at standard pressure of icosahedrite (Al_*x*_Cu_*y*_Fe_*z*_, with *x* between 61 and 64, *y* between 24 and 26, *z* between 12 and 13%)^29–31^.

**Figure 3:**
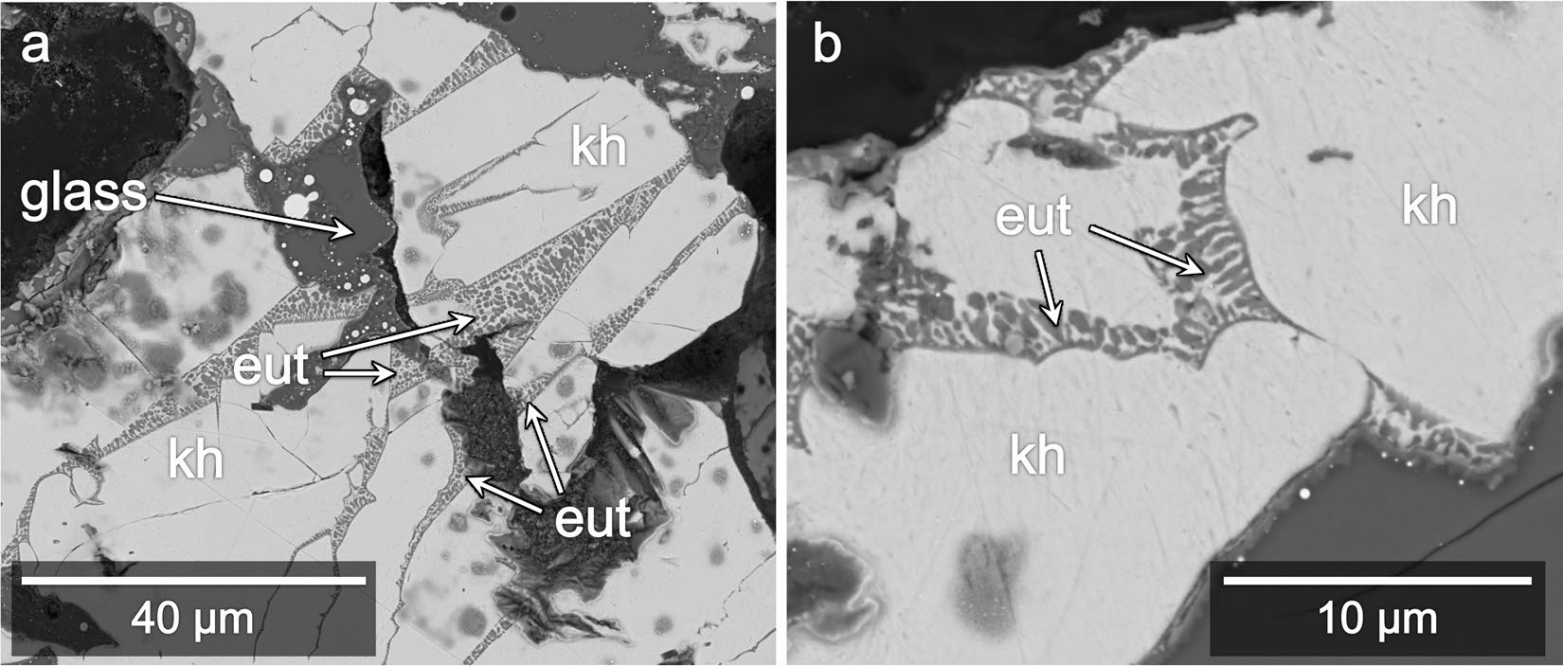
BSE images of eutectoid regions. (**a**) Close up of region overlapping with Locations 2 and 3 (Fig. 2a). The wedge-shaped silicate glass region appears to cross-cut khatyrkite metal (“kh”) and what may have been previously connected eutectoid regions (“eut”). (**b**) Close up of Location f.3b (Fig. 2a). Cuspate-lobate boundaries of the eutectoid regions here suggest they were partially molten, whereas the khatyrkite was solid.

The large (100–300 μm) Al-Cu-Fe metal fragments mostly comprise khatyrkite (CuAl_2_, with up to 2.68 elemental weight % Fe) and variable amounts of stolperite. Within some of the Al-Cu-Fe metal grains and sometimes along their edges, there are mixtures of khatyrkite and Al, with a vermicular texture (Fig. 3). The Al in these mixtures contains up to 13.3 elemental weight % Cu. Given their texture and composition, we refer to these regions as “eutectoid”. In Fig. 3a, the grain boundaries between glass and the metal alloys are relatively straight. Straight grain boundaries typically indicate formation by fracture or along crystal faces^32^. Here, we infer that the glass may have filled a fracture. The glass appears to cross-cut what may have been connected eutectoid regions. In Fig. 3b, the eutectoid regions have cuspate-lobate boundaries where they are in contact with the adjacent metal grains. Along these edges, the cusps tend to point away from the eutectoid regions.

**Figure 4:**
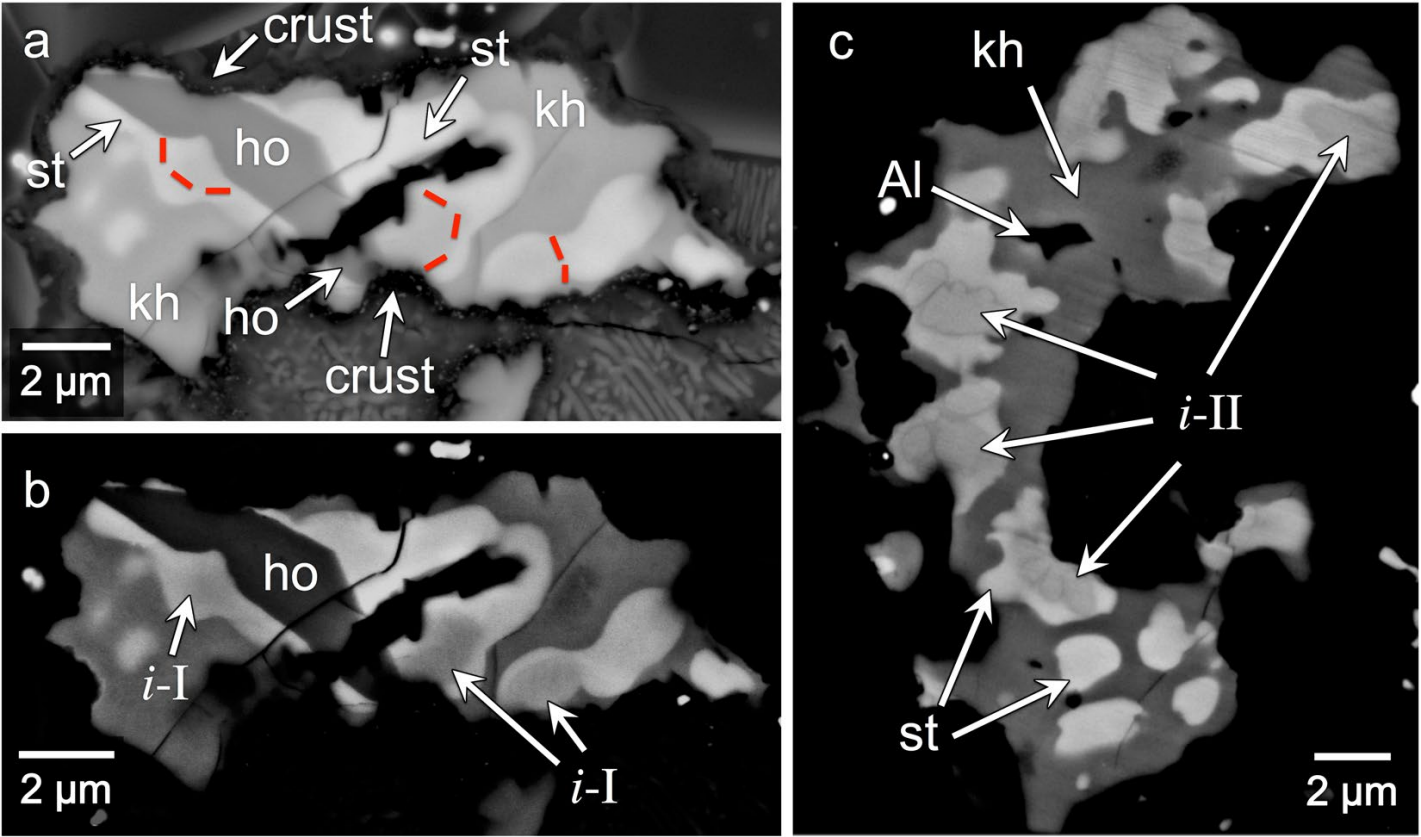
BSE images of Al-Cu-Fe assemblages with two generations of quasicrystals. Compositions for the metal phases shown here are listed in Table 1. Contrast in (**b**) and (**c**) has been stretched to emphasize compositional differences among the different metal phases. (**a**,**b**) Close up of Location 8 (Fig. 2a). This assemblage contains khatyrkite (“kh”), stolperite (“st”), hollisterite (“ho”), and icosahedrite (“*i*-I”). The red, dashed lines in (**a**) outline the icosahedrite grains to distinguish these grains from the neighboring phases; these sketches of the phase boundaries are based on higher contrast images of the same region, such as shown in (**b**). The upper and lower ends of the hollisterite grain terminate against the spinel crust (“crust”). We interpret this as a cross-cutting relationship that indicates the hollisterite and *i*-I are relict. The dark area in the center of the Al-Cu-Fe assemblage is a hole. (**c**) Close up of Location 10 (Fig. 2a). This assemblage contains quasicrystals (“*i*-II”) that are distinct from *i*-I. Surrounding *i*-II is stolperite, which is surrounded by khatyrkite. The dark area in the interior of this assemblage is metallic Al (“Al”). The dark area external to this assemblage contains silicate phases, which are not discernable in this image because of the high contrast.

There is a single occurrence of hollisterite (Fig. 4a), which occurs immediately adjacent to grains of *i*-I (Fig. 4b). The upper and lower ends of the hollisterite grain are overlaid by a crust of spinel crystals (see discussion of the spinel crust below). The grains of *i*-II are surrounded, sequentially, by stolperite, khatyrkite, and Al (Fig. 4c). Kryachkoite appears as euhedral grains within some eutectoid mixtures.

### Silicates and oxides

**Figure 5:**
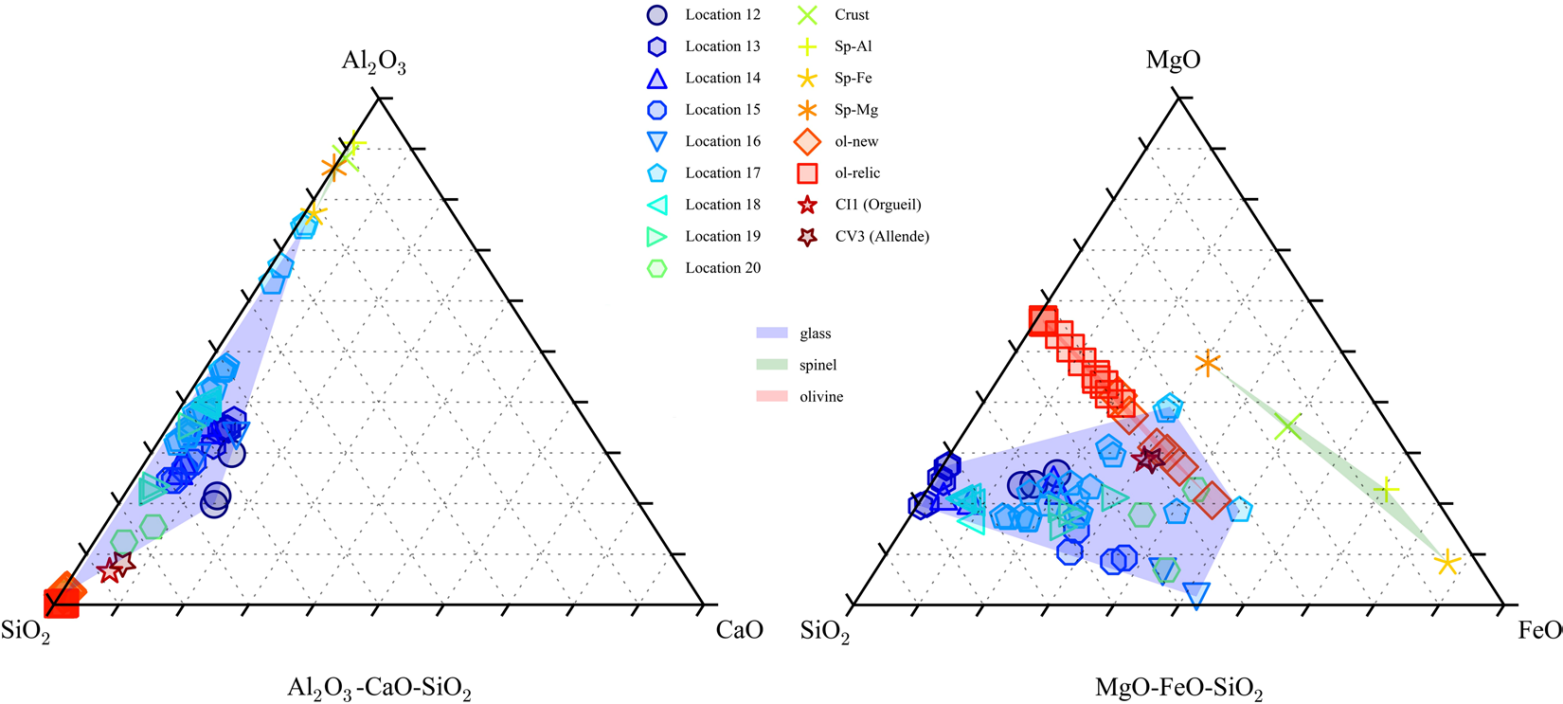
Compositions of glass regions in 126A. They are presented in terms of weight % Al_2_O_3_-CaO-SiO_2_ (left) and MgO-FeO-SiO_2_ (right) and normalized to 100%. The three shaded regions are the convex hulls of glass (blue), spinel (green), and olivine (red) compositions; each shaded region shows, roughly, the range of compositions for one of the three phases. Locations refer to those shown in Fig. 2b. Compositions vary widely both within the same region (see, e.g., Location 17) as well as across different regions. Also plotted for comparison are bulk compositions of the Orgueil (CI1) chondrite^59^ (Fe_2_O_3_ from original analyses has been recalculated to FeO) and of the Allende (CV3) chondrite^60^. Orgueil’s composition reflects bulk solar abundances, and Allende’s is prototypical of CV3 chondrites. Compared to the bulk compositions of Orgueil and Allende, the glass in Grain 126A contains higher relative abundances of Al_2_O_3_ and lower relative abundances of FeO. This is consistent with modification of the matrix melt (which may originally have had composition similar to that of Orgueil and Allende) by assimilation of oxidized Al following redox reaction with Al-Cu-Fe metal. During the reaction, the FeO from the matrix melt reduced to metallic Fe, which then formed Fe beads.

The material surrounding the Al-Cu-Fe metal grains is a fine-grained, hypohyaline assemblage of olivine and spinel quench crystals embedded in a glassy groundmass. Compositions taken from regions marked in Fig. 2b are presented in Table 2b. In Fig. 5, all of the compositions are plotted in the ternary diagrams in terms of Al_2_O_3_-CaO-SiO_2_ weight % and MgO-FeO-SiO_2_ weight %. The compositions are widely distributed, varying not only across different regions of the sample, but also within the same region.

Besides the glass, there are olivine crystals, contained primarily in the blue-boxed region in Fig. 2b that has been enlarged and shown separately in Fig. 2c. They exhibit a brecciated texture consisting of relatively larger (10–30 μm), fractured olivine grains, surrounded by fine-grained (2–10 μm) angular olivine fragments. Point analyses in this region are presented in Table 2a. All of the compositions are very close to stoichiometric olivine. The relatively larger grains have Mg-rich cores (Fo_77–99_). Their appearance and composition resembles the chondrule olivines found in Grains 5 and 121 of Khatyrka^2^. The relatively smaller grains have subhedral to equant euhedral morphology, more ferroan cores (Fo_61–73_) than the larger grains, and rims that are Fe-rich (Fo_45_ in S16, Table 2a). Some of the smaller grains are shown in Fig. 2d, an enlarged version of the red-boxed region in Fig. 2c.

**Figure 6:**
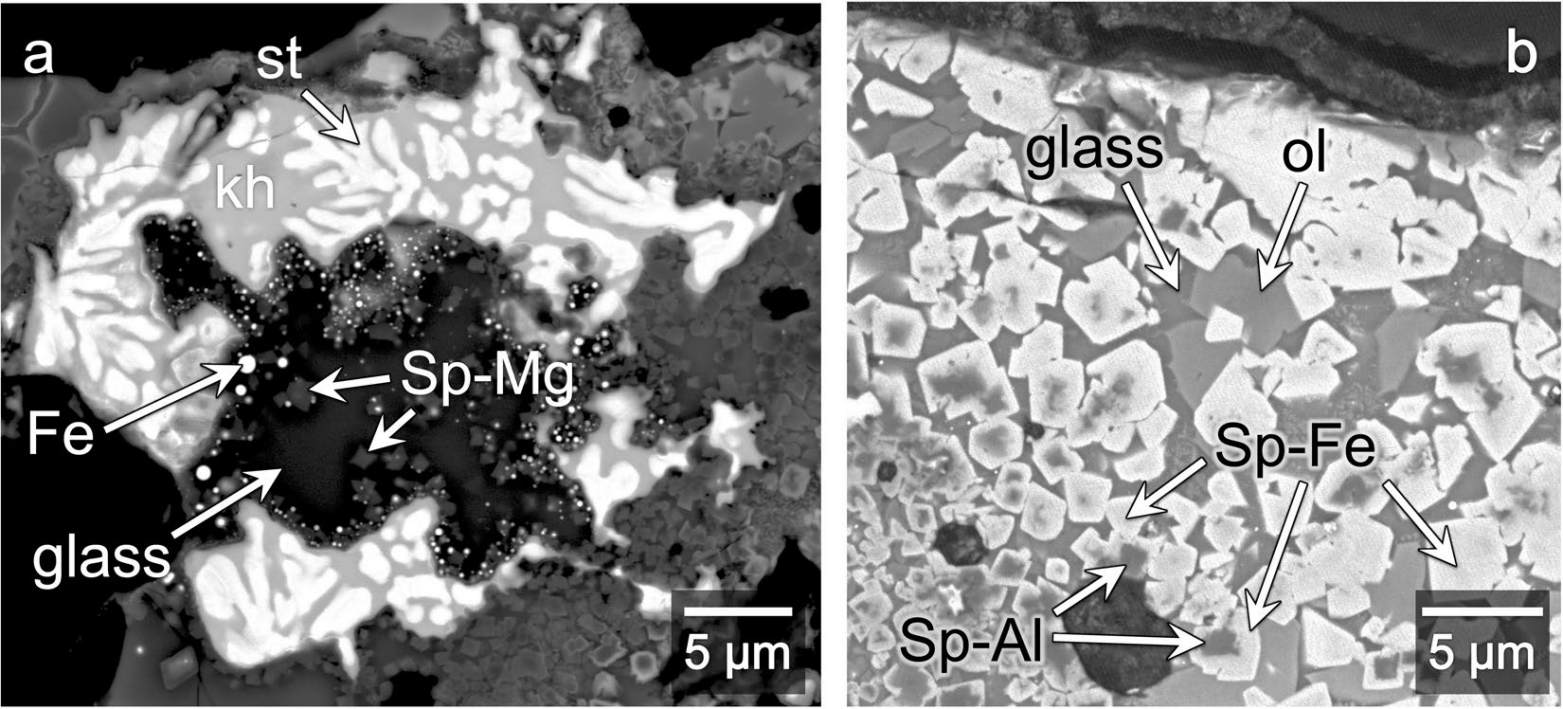
BSE images of various spinel phases. Compositions for spinel phases shown here are provided in Table 2c. (**a**) Close up of region overlapping with Locations 11 (Fig. 2a) and 18 (Fig. 2b). Khatyrkite (“kh”) and stolperite (“st”) have a texture indicating crystallization from a melt. Metallic Fe beads (“Fe”) line the interior interface. Spinel crystals that grew here in the silicate melt (now glass) are denoted as “Sp-Mg”. They have lower concentrations of FeO and Fe_2_O_3_ than the spinel crystals shown in (**b**). (**b**) Close up of Location 20 (Fig. 2b) showing Fe-rich spinel crystals (“Sp-Fe”) and zoned spinel crystals with Fe-rich rims (also marked “Sp-Fe”) and Al-rich cores (“Sp-Al”), all surrounded by glass. In contrast to (**a**), this region does not have Fe beads, but the spinel crystals here have higher concentrations of FeO and Fe_2_O_3_ than those in (**a**). Also noted in the image are olivine crystals (“ol”, Fo_50_-Fo_56_).

Averaged compositions of different spinel phases (Fig. 6) are presented in Table 2c. A layer of spinel crystals mantles each of the Al-Cu-Fe metal grains, separating the metal from the silicate regions (this is called “Crust” in Table 2c). This crust of spinel crystals forms part of the Al_2_O_3_-rich halo that envelops each of the metal grains (blue, Fig. 1b). Most metal grains also have a submicron-thin layer of Al-oxide located between the metal and the spinel crust. This Al-oxide layer is too thin to analyze for precise composition. In addition to the spinel crust, there are euhedral Mg-rich spinel crystals (~<2 μm; “Sp-Mg”, Fig. 6a, Table 2c). These spinel crystals tend to occur where the Al_2_O_3_ to FeO ratio in the adjacent glass is relatively high (more dark blue/purple, Fig. 1b), such as Locations 14, 17, and 18 (Figs 2b and 6a), where the observed weight % ratio of Al_2_O_3_ to FeO ranges from 1.09 to 13.95. There are also subhedral to equant euhedral spinel crystals, some that have Al-rich cores (“Sp-Al”, Fig. 6b, Table 2c) and Fe-rich rims (“Sp-Fe”, Fig. 6b, Table 2c), and others that are more homogeneous and near magnetite (Fe_3_O_4_) in composition (included in “Sp-Fe”, Fig. 6b, Table 2c). The Sp-Al and Sp-Fe phases occur where the Al_2_O_3_ to FeO ratio in the adjacent glass is relatively low (more green, Fig. 1b), such as Location 20 (Figs 2b and 6b), where the observed weight % ratio of Al_2_O_3_ to FeO ranges from 0.02 to 0.22.

**Figure 7:**
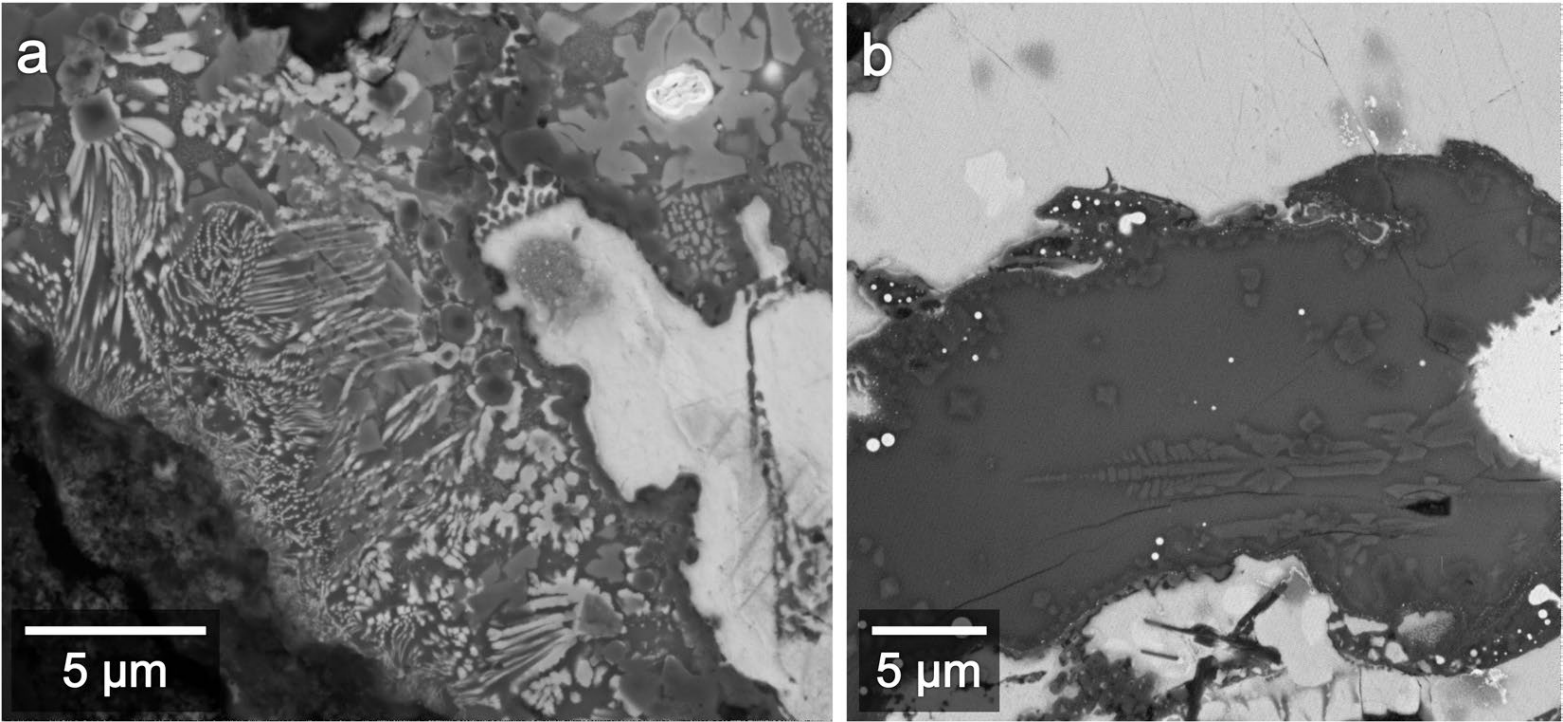
BSE images of skeletal, quench textures in the silicate glass. Individual phases are too small to analyze for precise compositions. (**a**) Close up of Location f.7a (Fig. 2b) showing granophyric textures. (**b**) Close up of Location 14 (Fig. 2b) showing feathery textures.

Lastly, there are silicate phases surrounded by glass that are too fine-grained to resolve analytically. They include, for example, skeletal, acicular crystallites and others that exhibit granophyric textures (Fig. 7).

### Metal beads and droplets (Fe, Fe-Si, Ni, Cu)

**Figure 8:**
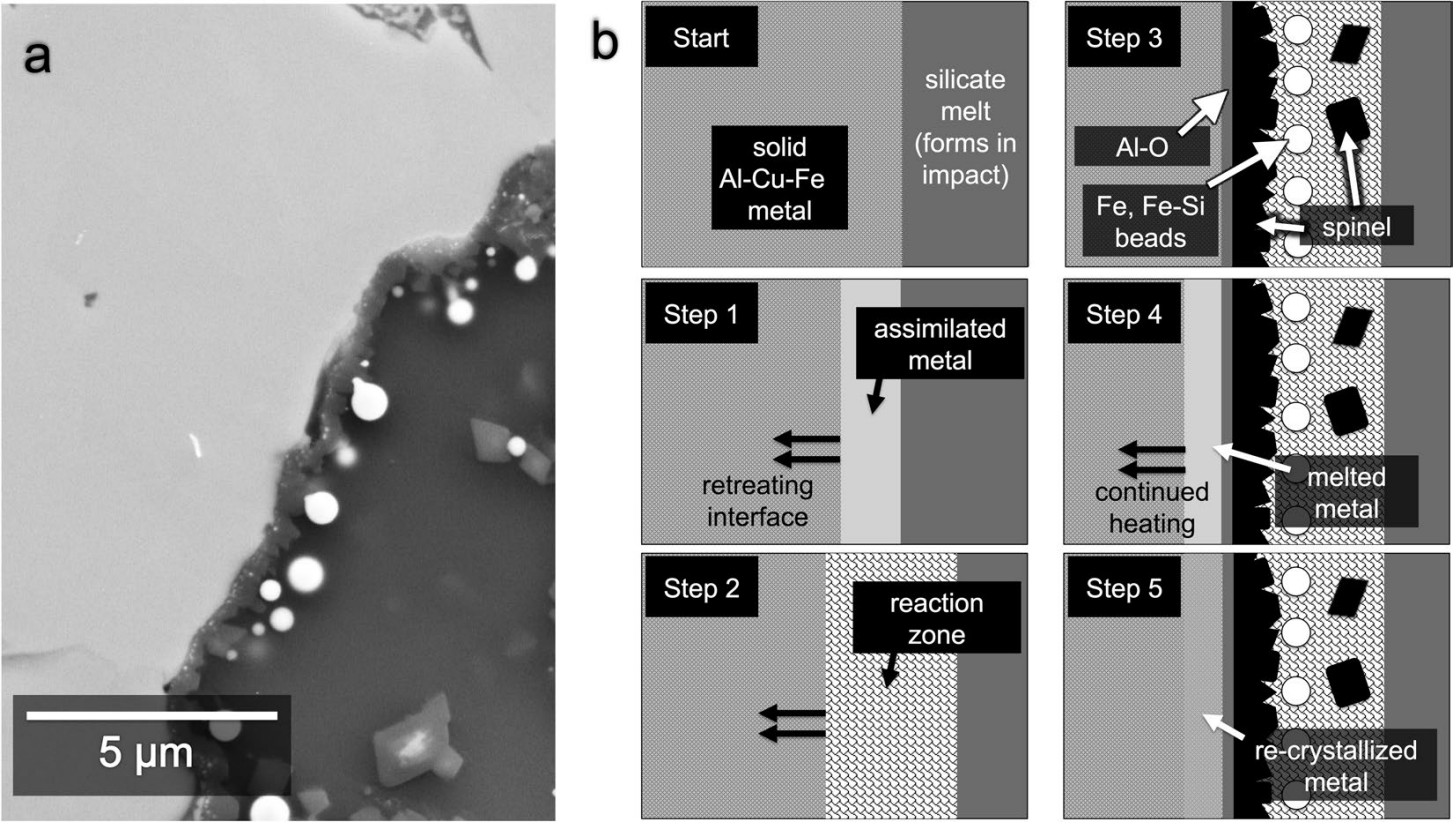
Redox reaction at metal-matrix interface. (**a**) BSE image showing close-up of Location f.8 (Fig. 2b). Metallic Fe beads occur mostly along interface between Al-Cu-Fe metal and glass. Spinel crust surrounds the Al-Cu-Fe metal grains. The Fe beads and spinel crust are the products of a redox reaction between Al-Cu-Fe metal and silicate melt. (**b**) Schematic illustration showing a proposed sequence of how the interaction unfolded between Al-Cu-Fe metal and silicate melt. The Start panel represents the initial contact between metal and silicate melt. In Step 1, the metal is being partially assimilated into the melt, which leads to an enrichment of Al, Cu, and Fe in the melt. The metal-matrix interface retreats into the metal. In Step 2, the interface continues to retreat, while the redox reaction occurs in the reaction zone, producing oxidized Al and metallic Fe and Si. In Step 3, the interface stops retreating and the redox reaction stops, as various phases begin to crystallize, including a layer of Al-oxide (“Al-O”), spinel crystals, and Fe and Fe-Si beads. In Step 4, the metal grains are still being heated. Some, like the one represented here, begin to melt, beginning at the metal-matrix interface. In Step 5, the system cools, and the newly melted metal regions re-crystallize. This final panel reflects what is observed in (**a**).

Near the interfaces between the Al-Cu-Fe metal grains and the silicates, there are spherical metallic Fe beads varying in size from less than 10 nm to ~5 μm (“Iron”, Table 1). Some of these beads are shown in Fig. 8a. The Fe beads contain variable amounts of Si, from below detection limit up to 35 weight %. Among the Fe-silicide beads are the minerals naquite (FeSi)^33^, suessite (Fe_3_Si)^34^ and xifengite (Fe_5_Si_3_)^35^ (Fig. 2a). Some of the larger Fe-beads contain S- and Cu-rich inclusions. The Fe- and Fe-Si beads are Ni-poor (as low as <0.1 weight %) and visibly distinct from the larger (3–25 μm) grains of taenite (“tae”, Table 1), which dominate the olivine field in the center-right of the sample. A 1 μm Ni-rich droplet (90 weight % Ni) and 3-μm large Cu-rich droplet (94 weight % Cu) were also identified; their compositions can be found in Table 1.

### Chemical reaction between Al-Cu-Fe metal and silicate matrix

Our study of Grain 126A shows that the melted silicate matrix and Al-Cu-Fe alloys reacted, probably exothermically, at temperatures above the bulk silicate solidus. Oxidized Fe and Si in the silicate melt were reduced by metallic Al in the adjacent alloy. In reciprocal fashion, the metallic Al was oxidized and incorporated into the silicate melt. Subsequently, the reduced Fe and Si coalesced into beads. The oxidized Al diffused into the adjacent silicate melt and formed an Al_2_O_3_-rich zone **(**Fig. 1b). Some of the oxidized Al formed the Al-oxide layer and the aluminous spinel that crystallized on the metal grains. This mantle of spinel sealed off the metal from further reaction.

The reaction products (Fe, Fe-Si beads, Al_2_O_3_ halo, Al-oxide layer, and spinel) are mostly present along the metal-matrix interfaces where the reaction would have initiated (see, e.g., Fig. 8a). This is the clearest observation yet of a reaction zone in Khatyrka. The further observation that these reaction zones are narrow, extending only tens of microns at most away from the metal, argues for an extremely rapid process. The observations here are qualitatively similar to what was found in the impact experiments reported in ref. 36; there, a metallic Al projectile (with minor Cu) was shot into a quartz sand target, resulting in a redox reaction that produced a reaction zone of Al_2_O_3_ and beads of metallic Si.

Metallic Fe and Fe-silicide beads in 126A are similar to ones described in Grain 129^21^. Such beads can be produced during impact^25, 33–35, 37–40^. The observation of metallic Si alloying with Fe further indicates that, during the impact, the conditions were sufficiently reducing to favor formation of Fe-Si phases over Fe + SiO_2_. However, there is no evidence that they were sufficiently reducing to produce Fe-Al phases in the silicate melt. We do, however, observe droplets of metallic Cu and Ni, consistent with these phases having *f*O_2_ requirements less stringent than even metallic Fe. The presence of Cu droplets implies the simplified reaction (Δ_f_ *H*° = −465 kJ, using parameters from refs 41–43):

$${{\rm{CuAl}}}_{2}+{{\rm{MgFeSiO}}}_{4}\to {\rm{Cu}}+{\rm{Fe}}+{\rm{Si}}+{{\rm{MgAl}}}_{2}{{\rm{O}}}_{4}.$$

(We have assumed here for simplicity that the silicate melt originally had a bulk composition similar to the porous matrix of the unshocked regions of Khatyrka^2^). In addition to Cu appearing in metallic droplets surrounded by glass, we observe Cu as a minor constituent in the glass (up to 1.4 weight % CuO).

The two varieties of olivine differ in both appearance and chemistry. The relatively larger olivine crystals (S1–S7, Fig. 2c, Table 2a) have no detectable P_2_O_5_ and, on average, no CuO. The smaller olivine grains (S13–S17, Fig. 2d, Table 2a) have 0.73–1.07 weight % P_2_O_5_ and 0.44–0.83% CuO. (There does not appear to be mixing of these analyses with neighboring glass, as there is no significant difference between concentrations of Al_2_O_3_, Na_2_O, and CaO in the cores (S13, Fig. 2d) and in the rims (S16 and S17, Fig. 2d).) The larger grains also have considerably less Al_2_O_3_: up to 0.17% Al_2_O_3_, compared with 0.69–1.12 Al_2_O_3_ in the smaller grains. For comparison, the neighboring glass has, on average, 1.63% P_2_O_5_, 0.80 CuO, and 18.42 Al_2_O_3_ (Location 16, Table 2b). From the appearance and chemistry, we infer that the larger olivine grains are pre-impact relics, whereas the smaller grains are a newer generation that grew rapidly from the P_2_O_5_- and CuO-bearing silicate melt. This interpretation is supported by experimental studies of P-rich olivine^44^ and nano-analytical studies of silicate inclusions in the Netschaëvo iron meteorite^45^.

### ‘Metal-forming event’ occurred prior to the ‘impact event’

As noted above, the cuspate-lobate boundaries between the Al-Cu-Fe metal grains and the glass have cusps that tend to point into the metal grains. The cuspate-lobate fold geometry forms as a buckling instability in compression, where the lobes point to the weaker material, and cusps point toward the stronger material^32^. Silicate melts have lower viscosities than solid metals, whereas in the solid-state metals are less viscous than silicates. We thus infer that the silicates were molten or partially molten when the cuspate-lobate grain boundaries formed. The texture is consistent with the Al-Cu-Fe metal grains being solid and the glass being a melt when they reacted to form the spinel crust and thin Al-oxide layer.

In Fig. 3a, the glass (formerly matrix melt) appears to cross-cut the eutectoid regions, which would indicate a time sequencing: the now-cut Al-Cu-Fe alloy existed before the fracturing and intrusion of the silicate melt.

The preceding observations suggest that the ‘metal-formation event’ occurred prior to and is distinct from the event that produced the silicate melt. This is further supported by the presence of cuspate-lobate eutectoid boundaries (Fig. 3b), which have a fundamentally different texture than the angular eutectoid regions (Fig. 3a). We infer from the difference in texture that two different processes generated them. The rounded boundaries are consistent with the khatyrkite adjacent to the eutectoid regions being incorporated into partially re-melted eutectic melt. Because these regions were partially re-melted, they must have first formed prior to the event that led to the partial re-melting.

### Two generations of Al-Cu-Fe quasicrystals

The upper and lower ends of the hollisterite grain terminate against an overgrowth of spinel crystals (Fig. 4a). We interpret this geometry to indicate a cross-cutting relationship that shows the hollisterite grain existed prior to the formation of the spinel layer, and, hence, the hollisterite grain is a pre-impact relict. This is further supported by the fact that the crust embays the contact between the hollisterite and stolperite where the arrow points to the crust in Fig. 4a. The icosahedrite grains lying immediately adjacent to the hollisterite (see Fig. 4b) must also be relicts, and, thus, the icosahedrite grains are older than those formed during the most recent impact a few 100 Ma.

The *i*-II-bearing assemblage (Fig. 4c) occurs ~50 μm to the right of the hollisterite-bearing assemblage. The metal grains are similar in size (~15 μm). However, the *i*-II-bearing assemblage does not contain either hollisterite or *i*-I. Moreover, the textures in the *i*-II-bearing assemblages strongly suggest that these assemblages are not relicts but, rather, had solidified from completely re-melted metal following the ‘impact event’. The enclosed phases, including the *i*-II phase, formed during a new crystallization sequence: the *i*-II phase crystallized first from the Al-Cu-Fe liquid; subsequently, the stolperite grew around the *i*-II phase, shielding the residual Al-Cu-Fe liquid from the *i*-II phase; within this residual liquid, the khatyrkite crystallized from a peritectic reaction. Finally, at the eutectic point, khatyrkite and Al formed together. The age of the *i*-II phase would thus be a few 100 Ma (the time of the latest impact).

Although the *i*-II-bearing assemblage and the hollisterite-bearing assemblage are similar in size and in close proximity to one another, they are chemically distinct. As discussed in ref. 14, these differences may be due to the highly non-equilibrium conditions experienced by Khatyrka due to impact. The *i*-II phase may be a kinetically stable but thermodynamically unstable phase, which has been preserved due to the quench. These two quasicrystal-bearing assemblages share a resemblance to those obtained in the experiments reported in Figs 13 and 15 of ref. 46.

With the *i*-I grains having formed prior to the impact event and the *i*-II phase having formed after, we can make the following conclusions: first, there are at least two generations of quasicrystals in Khatyrka; second, Khatyrka experienced conditions suitable for producing quasicrystals both prior to and during the ‘impact event’; and, third, the Al-Cu-Fe metal assemblages experienced different degrees of melting during the ‘impact event’, with some completely melting (like the *i*-II-bearing assemblage, Fig. 4c), some only partially melting (like the eutectoid region in Fig. 3b), and others not melting at all (like the relict eutectoid in Fig. 3a).

### Rapid cooling, heterogeneous temperature distribution, and new temperature constraints

The highly heterogeneous composition of the glass (as seen in the ternary diagrams in Fig. 5) and the presence of skeletal crystals (Fig. 7) indicate that the silicate melt cooled rapidly enough so that there was not enough time for compositional variations to be smoothed out by diffusion. Hence, 126A experienced at least two stages of extreme thermal changes caused by the latest impact event: first, a period of rapid heating, initiated by the impact and assisted by the aluminothermic reaction; and, subsequently, a rapid quench.

The effects of impact are heterogeneous at the grain scale. We can obtain local constraints—not on the entire body but for particular spots—on the temperature attained during the ‘impact event’ by identifying phases that did not fully melt. For these temperature constraints, we assume ambient pressures—even though high-pressure phases were found in other fragments of 126 and in other grains of Khatyrka.

The presence of relict chondritic olivine implies that the meteorite was not 100% melted during the ‘impact event’. Specifically, the presence of relict Fo_77_ implies that the peak temperature in these relict cores during the ‘impact event’ was no higher than ~1,700 °C, the melting temperature of Fo_77_ at atmospheric pressure^47^. As discussed previously, the appearance and composition of these relict olivine grains is similar to the chondrule olivines observed elsewhere in Khatyrka^2^.

Where the olivine was partially melted, we can infer that those regions were hot enough to melt olivine and pyroxene; hence, they were heated to at least 1,200 °C, which is set by the solidus in the Fa-Fo-SiO_2_ ternary^47^. This latter estimate is corroborated by the presence of Fe-silicide beads. The crystallization of these beads from the silicate melt implies that they experienced temperatures as high as 1,200 °C, about the temperature of the solidi in the Fe-Si binary system^48^. This temperature estimate is also consistent with that obtained in ref. 22 from an interpretation of ladder-like veins of ahrensite + SiO_2_.

The Al-Cu-Fe metal yields a much lower peak temperature estimate, based on the relict eutectoid regions containing Al and khatyrkite. The cross-cut eutectoid regions (Fig. 3a) were heated to at most ~540 °C, the Al-khatyrkite eutectic temperature^49^. If the peak temperature were higher, these regions would have completely re-melted and the cross-cutting relationship would not be preserved.

The very different peak temperature estimates from the relict metal eutectoid regions and the partially melted olivine regions, 540 °C and 1,200 °C respectively, reinforce the earlier conclusion that cold, solid metal was in contact with hot, molten matrix material during the ‘impact event’. This also presents stark evidence for a heterogeneous distribution of temperatures in 126A following the ‘impact event’.

We can also constrain the maximum peak temperatures for the hollisterite grain and the adjacent icosahedrite. As discussed previously, the cross-cutting relationship with the spinel crust indicates that these phases are relict. If these phases were heated above their melting temperatures after the onset of the aluminothermic reaction, they would have melted and the cross-cutting relationship would not be preserved. We conclude that the hollisterite and icosahedrite grains were heated to at most around 1,160 **°**C and 1,100 **°**C, their respective melting temperatures^29, 30, 50^.

In those regions containing stolperite (e.g., the quasicrystal-bearing assemblages and the region shown in Fig. 6a) we can constrain the temperature at the time of quench, which may be different from the peak temperature. We interpret the texture as crystallization from a melt, with the stolperite grains appearing to be partially resorbed. The presence of stolperite implies that the temperature of the metal when it was quenched was not higher than the melting temperature of stolperite, which is around 640 °C ^46^.

## Discussion

We assume for the purposes of simplicity that a single shock event is associated with the production of the high-pressure phases in 126 and in other grains, as well as with the silicate melt and redox reaction observed in 126A. Features in 126A and other samples indicate that this shock event produced a highly heterogeneous range of temperatures and pressures, followed by rapid cooling. Our interpretations are corroborated by the studies in refs 18 and 19 of noble gas measurements on forsteritic olivine fragments of Grain 126 (not from 126A) that show a high-velocity impact event occurred a few 100 Ma. This impact event resulted in shock stages ranging from S3 to S5 but most likely around S4^25^.

The textural relationships and evidence of redox reaction found in 126A indicate that some of the Al-Cu-Fe alloy phases (including quasicrystals) formed prior to the impact event and some during, firmly establishing that the Al-Cu-Fe alloys (including icosahedrite) are extraterrestrial in origin and have a shared history with the chondritic material in outer space. While the noble gas studies indicate that the formation of the Al-Cu-Fe alloys occurred a few 100 Ma, the formation of the first Al-Cu-Fe alloy phases was probably no earlier than 4.564 Ga based on the absence of any evidence of excess ^26^Mg (radiogenic from the decay of the short-lived nuclide ^26^Al) in any of the measured Al-bearing metal grains from Khatyrka^1, 2^. (However, it is possible that any excess ^26^Mg was reset during the impact event a few 100 Ma).

Our study also shows that the ‘single event hypothesis’ of ref. 22 can be ruled out. In this hypothesis, the ‘metal-forming event’ and ‘impact event’ are the same. Although this hypothesis was favored for its simplicity, the results in this paper show that a more complex multiple-stage process is necessary to explain the observations: at the very least, a ‘metal-forming event’ was followed by one or more later ‘impact-events,’ with the most recent major impact occurring a few 100 Ma. Furthermore, the original ‘metal-forming event’ must have entailed highly reduced conditions, at least below the (CuO + Al_2_O_3_)/CuAl_2_ buffer, in order to form khatyrkite. This makes it especially challenging to formulate a reasonable theory for how the Al-Cu-Fe metals first came to be, and is thus an ongoing question for researchers. Speculations include: (i) condensation from the presolar nebula; and (ii) a carbon-mediated extraction process (i.e., smelting), which could take place, for example, upon extreme heating of neighboring Cu- and Al-rich phases within ureilite-like carbon-rich material (see refs 51 and 52 for further discussion on such a reduction mechanism).

One of the striking features of 126A reported here is the presence of unmelted Al-Cu alloy, with melting temperature ~540 °C ^49^, in contact with silicate glass, with melting temperature ~1,200 °C ^47^. We note that ref. 53 observed similar textures to what we observe (Fig. 1) with respect to Fe-Ni metals in contact with silicates, in a study of three CB carbonaceous chondrites (Queen Alexandra Range (QUE) 94411, Hammadah al Hamra 237, and Bencubbin). One example, within QUE 94411, is a relict grain of Fe-Ni embedded in a shock melt (see Fig. 7 of ref. 53). They argue that the precursor to the shock melt was fine-grained porous matrix material, and that, during a shock event, differences in shock impedance between denser, non-porous Fe-Ni metal and less dense, porous silicate matrix led to localized melting in the matrix material, which formed the now-observed shock melt. (See refs 25 and 54 for further discussion of disequilibrium shock effects). The features observed in Grain 126A, including the contact between cold metal and silicate melt, can also be interpreted as the effects of highly localized, heterogeneous, shock-induced melting as a result of fine-scaled shock impedance variation. The main difference from the study of ref. 53 and ours is that instead of relict Fe-Ni metal with melting temperature ~300 °C^55^, Grain 126A contains the relatively higher melting-point Al-Cu(-Fe) alloys.

We suggest that, after the silicate melt formed, the melt interacted with the Al-Cu-Fe metal grains as follows (see Fig. 8b for a schematic illustration): The start panel in Fig. 8b represents the initial contact between silicate melt and cold metal. (Step 1) The Al-Cu-Fe metal, beginning at the grain boundaries, was partially assimilated into the melt. The metal-matrix interface retreated into the metal (indicated by arrows, Fig. 8b), leaving behind a modified matrix melt (“assimilated metal”, Fig. 8b) with elevated concentrations of Al, Cu, and Fe. (Step 2) While the metal-matrix interfaces continued to retreat, the kinetic and chemical conditions were eventually suitable for ignition of an aluminothermic reaction near the interfaces, whereby Al was oxidized and Fe and Si were reduced (“reaction zone”, Fig. 8b). (Step 3) The redox reaction and the retreat of the metal-matrix interface stopped. A layer of oxidized aluminum and a crust of aluminous spinel crystals grew on the metal grains into the modified matrix melt. Fe and Fe-silicide beads formed within the modified matrix melt, mostly along the metal-matrix interface, and alongside spinel crystals. These phases are indicated in Fig. 8b. At this stage, the Al-Cu-Fe metal grains—including those enclosed by a crust of spinel crystals—were still solid but continued to be heated. (Step 4) The metal grains heated to varying degrees. Some did not melt, others partially melted, and others completely melted. Represented in Fig. 8b is a melting grain, with the melt front advancing into the metal. (Step 5) Where there was newly melted metal encapsulated by the spinel crust, a new crystallization sequence occurred (“re-crystallized metal”, Fig. 8b).

Of the quasicrystals in the Khatyrka meteorite, some like *i*-II formed during the latest ‘impact event’. Therefore, it appears that the conditions leading to the formation of quasicrystals were replicated in the ‘impact event’. Shock was also involved in the experiment reported in ref. 56, which was an attempt to simulate the starting materials and shock conditions experienced by Khatyrka and which resulted in the synthesis of another novel quasicrystal. We note that, while the shock may be a sufficient condition for the formation of natural quasicrystals, it may not be necessary.

The new observations given in this paper firmly establish that the quasicrystals and other metallic Al-bearing alloys in Khatyrka formed in outer space. We expect that further examples of exotic metal alloys eventually will be discovered as new and different types of meteorites are found and studied. The question of how the metallic Al was first reduced and then alloyed with Cu remains unanswered, and is the primary focus of ongoing investigations.

## Methods

### Sample characterization techniques

The sample studied here (Grain 126A) was first embedded in epoxy resin, prepared as a polished thick section, and coated with a 30-nm-thick carbon film. The results included here are from SEM-EDS (scanning electron microscopy, energy dispersive spectroscopy) and EPMA-WDS (electron microprobe microanalysis, wavelength dispersive spectroscopy).

### Scanning electron microscopy

X-ray compositional maps were obtained at the Centre for Electron Microscopy and Microanalysis (MEMA) of the University of Florence, Italy, using a Zeiss EVO MA15 SEM coupled with an Oxford INCA250 energy-dispersive spectrometer, operating at 25 kV accelerating potential, 500 pA probe current, and acquisition times of 10 ms per pixel. Further SEM-EDS studies were performed at Princeton University’s Imaging and Analysis Center and at the Smithsonian Institution. Most of the SEM-EDS compositions presented in this paper were obtained at the GPS analytical facility at Caltech, using a GPS ZEISS 1550VP field emission SEM equipped with an angle-sensitive back-scattered electron (BSE) detector, an Oxford X-Max SDD EDS, and an HKL EBSD (electron backscatter diffraction) system. BSE imaging, EDS and EBSD analyses were conducted using SmartSEM, AZtec and Channel 5 software. EBSD analyses at a sub-micrometer scale were performed at 20 kV and 6 nA in focused beam mode with a 70° tilted stage and in a variable pressure mode (25 Pa), using methods described in refs 57 and 58. The sample was vibro-polished to remove the carbon coat prior to EBSD analysis.

### Electron microprobe

Quantitative elemental microanalysis was carried out at the GPS analytical facility at Caltech, using a JEOL 8200 electron microprobe (WDS mode, 12 kV and 5 nA for metals, 15 kV and 10 nA for silicates and oxides, focused beam). Counting times were 20 s on-peak and 10 s each on upper and lower background positions. Data reduction used the CITZAF routine built into the Probe for EPMA software. Standards used for metals were: pure metal standards (Al, Si, Cr, Fe, Ni, Cu), forsterite (Mg) and anorthite (Ca). Standards used for silicate, oxide, and glass phases were: forsterite (Si, Mg), fayalite (Fe), albite (Na), microcline (K), anorthite (Ca, Al), Cr_2_O_3_ (Cr), NiO (Ni), apatite (P) and metal-Cu (Cu). For the spinel regions, it was difficult to obtain clean analyses of the small individual crystals. This resulted in weight percent totals for the spinel phases that deviated by several percentage points from the ideal 100% and spinel analyses that regularly contained >1 weight % SiO_2_, which is apparently from glass and/or olivine from the surroundings or from underneath. In the spinel compositions presented in Table 2c, we have included in the average only individual analyses whose weight % totals were between 90 and 110%. The total shown in the rightmost column is the averaged total.
